# Supplementary material for: Homeostasis at different backgrounds: The roles of overlayed feedback structures in vertebrate photoadaptation
Source: PLoS One. 2023 Apr 28;18(4):e0281490. doi: 10.1371/journal.pone.0281490 (PMC10146485; doi:10.1371/journal.pone.0281490)
Supplement: S1 File — (part 1). A zip-file with python scripts describing the results for motifs m1 (Fig 4a), m7 (Fig 6a), m2 (Fig 8a), m8 (Fig 11a), m3 and m5 (S1 Text, Figs S2a and S4a), m4 (S2 Text, Fig S2), and m6 (S2 Text, Fig S4a). (part 2). A zip-file with python scripts describing the results for Figs 15, 16a, 16b, 17b and 19. (ZIP) [file pone.0281490.s001.zip › S1 Programs part1.zip!/m2 python/plots/A_plots_merged.pdf]

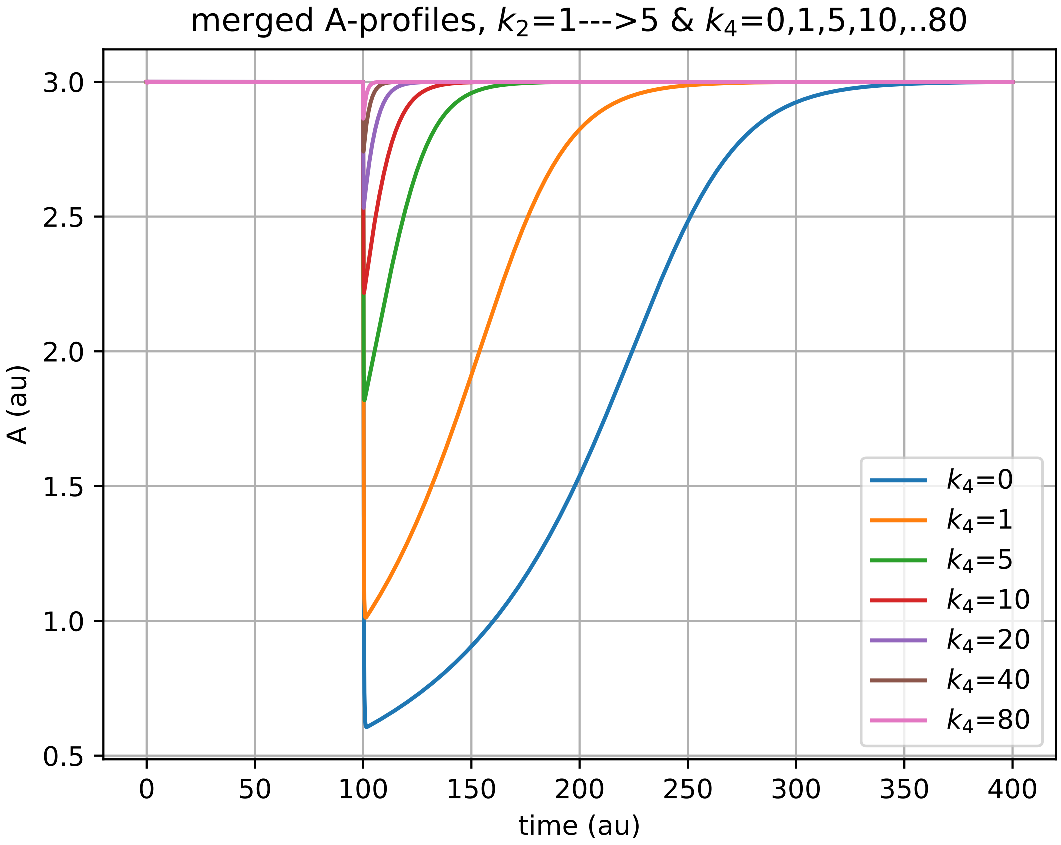
merged A-profiles, k_2=1--->5 & k_4=0,1,5,10,..80
A (au)
time (au)
3.0
2.5
2.0
1.5
1.0
0.5
0
50
100
150
200
250
300
350
400
k_4=0
k_4=1
k_4=5
k_4=10
k_4=20
k_4=40
k_4=80
